# Syphilis Screening in Correctional Facilities — Alaska, October 2023–September 2025

**DOI:** 10.15585/mmwr.mm7536a1

**Published:** 2026-09-17

**Authors:** Elizabeth Ohlsen, Julia Rogers, Sara Clark, Robert Lawrence, Joseph McLaughlin

**Affiliations:** 1 Alaska Department of Health, Anchorage, Alaska.

**Summary**

**What is already known about this topic?**

Since 2017, syphilis cases have increased substantially in the United States, including in Alaska. Historically, syphilis prevalence has been high among incarcerated populations.

**What is added by this report?**

During October 2023–September 2025, opt-out syphilis screening (i.e., routine testing unless a person declines) in Alaska correctional facilities found reactive rapid plasma reagin (RPR) results among 4.6% of screened persons, including 9.3% of women screened and 3.5% of men screened. These findings indicate high syphilis rates among incarcerated Alaskans who received testing. Reactive RPRs often indicate a past or current syphilis infection. Among all 5,163 persons screened, 2.2% had newly identified syphilis; of these, 77.2% completed syphilis treatment during this period.

**What are the implications for public health practice?**

Syphilis testing and treatment in correctional facilities might increase syphilis detection and treatment among persons who are incarcerated or detained, reduce community syphilis transmission, and help prevent congenital syphilis.

## Abstract

Since 2017, syphilis cases have increased substantially across the United States, including in Alaska. Nationwide, prevalence of syphilis tends to be higher among incarcerated persons than among the general population. During 2023–2025, opt-out screening for syphilis in Alaska correctional facilities (i.e., routine testing unless a person specifically declines) found reactive rapid plasma reagin (RPR) results, which often indicate a past or current syphilis infection, in 240 (4.6%) of 5,163 persons screened. Among persons with reactive RPR results, 114 (47.5%) had newly identified syphilis cases (2.2% of all persons screened), based on the 2018 Council of State and Territorial Epidemiologists definition. Among persons with newly identified syphilis, 88 (77.2%) were known to have completed treatment by September 2025. Among 1,020 women incarcerated in Alaska who were screened (19.6% of all persons screened), 95 (9.3%) had reactive RPR results. Among those 95 persons, 37 (3.6% of women screened) had newly identified confirmed or probable syphilis, and the remaining 58 (5.7% of women screened) had positive treponemal test results, demonstrating a high current or past syphilis prevalence among women incarcerated in Alaska. Implementing opt-out screening and treatment in correctional facilities could improve syphilis detection and treatment among persons in correctional facilities, and thereby reduce community syphilis transmission and help prevent congenital syphilis.

## Introduction

Syphilis is a sexually transmitted bacterial infection caused by *Treponema pallidum.* Syphilis during pregnancy can result in congenital syphilis, which can lead to severe illness and death in a fetus or infant (*1*). Rapid identification and treatment of persons with syphilis and testing and treatment of their sex partners can prevent transmission (*2*). Reducing community syphilis rates and ensuring universal syphilis screening and timely treatment during pregnancy are critical to preventing congenital syphilis (*2*).

### Syphilis in Alaska

From 2016 to 2024, the number of reported syphilis cases in Alaska increased almost 2,200%, from 20 to 452 (61 cases per 100,000 population), amid a nearly 700% national rise in total and congenital syphilis cases (*3*,*4*). Among the 452 syphilis cases reported in Alaska in 2024, nearly one half (216; 47.8%) were in women, most of whom (197; 91.2%) were of reproductive age (15–44 years) (*3*). Reported cases of congenital syphilis in Alaska increased concurrently, with zero cases as recently as 2019, to a record annual high of 12 cases in 2022, corresponding to 128 cases per 100,000 live births (*3*). Since then, 10 cases were reported in 2023 (105 per 100,000 live births), and six in 2024 (66 per 100,000 live births).

### Syphilis in Incarcerated Persons

Nationally, rates of syphilis among incarcerated persons have been higher than those in the general population (*5*). Amid this historic increase in syphilis and congenital syphilis in Alaska, and as part of a statewide effort to reduce congenital syphilis, on October 16, 2023, the Alaska Department of Corrections (AKDOC), in partnership with the Alaska Department of Health (AKDOH), initiated opt-out screening (i.e., universal routine testing on admission, unless a person specifically declines) for syphilis in correctional facilities, a strategy recommended in CDC guidelines to increase testing rates and reduce associated stigma (*6*). CDC recommends conducting opt-out syphilis screening for incarcerated persons based on the local and institutional prevalence of infectious syphilis.

## Methods

### Syphilis Screening

Beginning in October 2023, adults in Alaska who were detained or incarcerated were screened for syphilis, HIV, gonorrhea, chlamydia, and hepatitis C on an opt-out basis during a routine medical visit conducted within 14 days of their arrival at the facility. A presumptive diagnosis of syphilis requires use of two laboratory serologic tests: a nontreponemal test and a treponemal test. Syphilis screening at AKDOC followed the traditional screening algorithm (*6,7*), beginning with a rapid plasma reagin (RPR) test, a nontreponemal test that detects lipoidal antibodies that, when present, often indicate a current or past syphilitic infection.* If the nontreponemal test is reactive (i.e., detection of nonspecific antibodies), the specimen is diluted to obtain a titer, and a treponemal test (specific to syphilis) is performed on the same sample to confirm syphilitic infection (Supplementary Figure 1). When results were suggestive of syphilis, case investigation and partner services were initiated per AKDOH syphilis disease intervention practices (Tomas Hernandez, AKDOH, unpublished information, 2024). This project was reviewed by the AKDOH Scientific Review Committee and deemed nonresearch public health surveillance.

### Data Sources and Analysis

Laboratory results were obtained from electronic reports of screening tests performed on persons in Alaska correctional facilities during October 16, 2023–September 12, 2025. AKDOC facilities are for adults and serve as both jails (which house persons pretrial or for a short sentence) and prisons (for persons convicted of an offense for which they have been sentenced to a longer term of incarceration); juvenile facilities are administered separately through the Alaska Division of Juvenile Justice and are not included in these data. All AKDOC facilities were included in these data. Demographic and clinical information including age, sex, pregnancy status, previous syphilitic infection history, identification of syphilis stage, and syphilis treatment history were obtained from clinician reporting and public health surveillance records. The Alaska Section of Epidemiology surveillance database was queried for investigations associated with AKDOC facilities. Information was not available on the number or characteristics of persons who opted out of syphilis screening upon entering AKDOC custody.^†^ Descriptive analyses were performed using RStudio (version 2026.03; R Foundation).

### Case Definitions

Syphilis cases were identified and staged using the 2018 Council of State and Territorial Epidemiologists (CSTE) definition (*8*) and treated according to CDC guidelines (Supplementary Figure 2) (*6*). Cases were reported to AKDOH per state statutory reporting requirements and to CDC per federal requirements.

**Case identification.** Persons were categorized as having a reactive RPR test result if at least one reactive RPR result was documented while in AKDOC custody during the opt-out period (October 16, 2023, through the date of surveillance database query on September 12, 2025); this categorization included persons who also had one or more nonreactive RPR result in addition to the reactive RPR result. Persons with repeat or persistent syphilitic infections during the opt-out period, ascertained by case investigation, were counted once in these data. Persons with only nonreactive RPR results (and no reactive RPR result) during the opt-out period were categorized as not having a reactive RPR. Persons with a reactive RPR result also received treponemal testing and were considered to have a newly identified syphilis case if they met CSTE criteria for probable or confirmed syphilis and this infection had not been previously identified (i.e., the person had not previously reported syphilis, or the 2018 CSTE syphilis surveillance case definition was newly met based on RPR titer increase or signs or symptoms of primary or secondary syphilis).

**Documentation of treatment.** Persons with syphilis were considered to have been fully treated if it was documented that they received appropriate treatment for their stage of infection, which for these persons was a single dose of benzathine penicillin or a 14-day course of doxycycline for primary, secondary, or early nonprimary nonsecondary syphilis, or 3 doses of benzathine penicillin or 28 days of doxycycline for unknown duration or late syphilis, per national guidelines (*6*). Partial treatment was defined as documentation of receipt of at least 1 dose of benzathine penicillin or doxycycline, without meeting criteria to be considered fully treated.

## Results

### Participant Characteristics and Syphilis Screening Results

During October 2023–September 2025, a total of 5,163 adults were screened for syphilis in Alaska correctional facilities; the median age was 35 years (range = 18–83 years) and 4,141 (80.2%) were men (Figure) (Table 1). Overall, 240 (4.6%) persons received reactive RPR results, including 95 (9.3%) of 1,022 women, 88 (92.6%) of whom were considered to be of reproductive age (18–44 years); and 145 (3.5%) of 4,141 men. No false positive results were identified.

**FIGURE:**
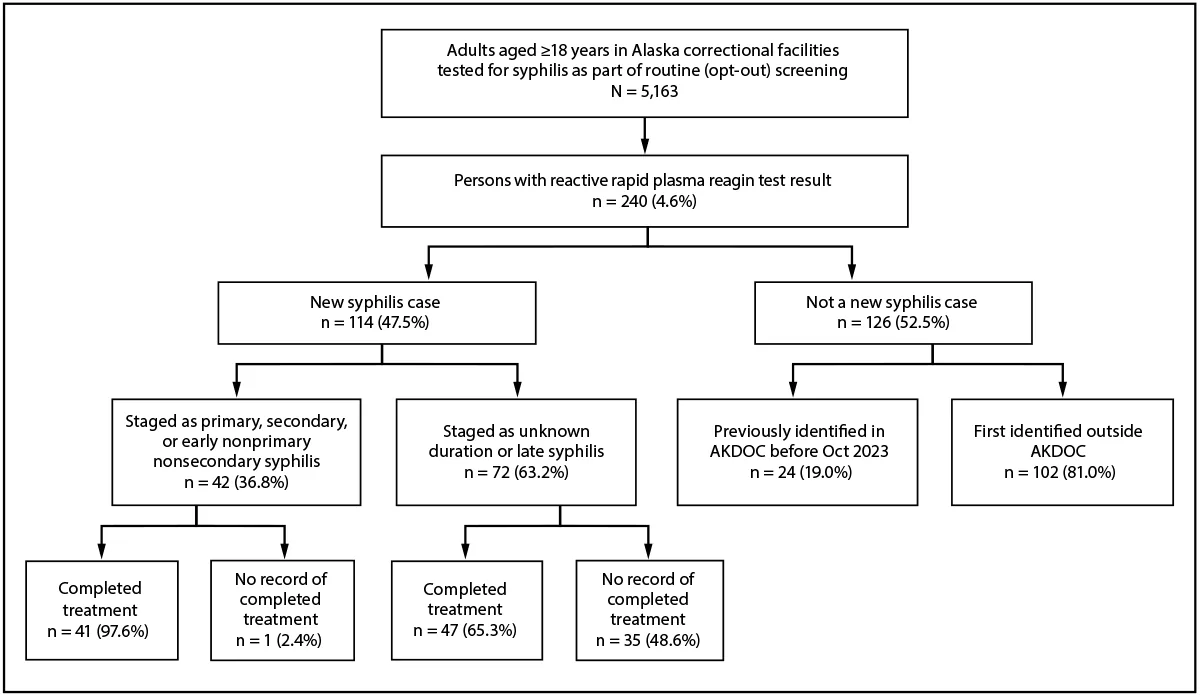
Results of syphilis screening with rapid plasma reagin tests and syphilis treatment status among persons in correctional facilities* — Alaska, October 2023–September 2025 **Abbreviation**: AKDOC = Alaska Department of Corrections. * Percentages are calculated using the number in the preceding box as the denominator.

**TABLE 1:** Results of syphilis screening with rapid plasma reagin tests among persons in correctional facilities, by age and sex — Alaska, October 2023–September 2025

Characteristic*	Total persons screened, no. (column %)	RPR test result,* no. (row %)	
		≥1 reactive RPRs	No reactive RPR^†^
Total*	**5,163 (100.0)**	**240 (4.6)**	**4,923 (95.4)**
**Sex**			
Female	1,022 (19.8)	95 (9.3)	925 (90.7)
Male	4,141 (80.2)	145 (3.5)	3,996 (96.5)
**Age, yrs**			
Median	35	36	35
(range) [IQR]	(18–83) [28–41]	(19–66) [29–41]	(18–83) [28–42]

**Abbreviation:** RPR = rapid plasma reagin.

* No other demographic information was available.

^† ^RPR testing detects lipoidal antibodies; when present, they often indicate a current or past syphilitic infection. If the test is reactive (i.e., detects nonspecific antibodies), the RPR specimen is diluted to obtain a titer, and a treponemal test specific to syphilis is performed on the same sample to confirm syphilitic infection.

^§^ A person was counted only once regardless of the number of times screened. If at least one RPR test result was reactive, the person was considered to have a reactive RPR.

Among the 240 persons with reactive RPR results, 114 (47.5%) met criteria for a new case of probable or confirmed syphilis (Table 2) according to the CSTE case definition. These accounted for 2.2% of all persons screened (corresponding to an estimated prevalence of 2,208 per 100,000 persons screened [95% CI = 1,841–2,648]). Among the 88 women of reproductive age with a reactive RPR, 37 (42%) had newly identified syphilis. Among these 37 women, pregnancy status was known for 32 (86%), one of whom was pregnant at the time of screening. The most common reason for unknown pregnancy status was having declined pregnancy testing at the time of clinical services and at the time of public health outreach.

**TABLE 2:** Syphilis cases and treatment among persons with reactive rapid plasma reagin* test results in correctional facilities — Alaska, October 2023–September 2025

Characteristic*	No. (%)^†^
**Reactive treponemal test result**	**240 (100.0)**
Sex	
Female	95 (39.6)
Reproductive age (15–44 yrs)^§^	88 (36.7)
Male	145 (60.4)
Syphilis case status	
New case	114 (47.5)
Not a new case, identified in AKDOC before Oct. 2023	24 (10.0)
Not a new case, first identified outside AKDOC^¶^	102 (42.5)
**Persons with newly identified syphilis**	**114 (100.0)**
Stage	
Primary, secondary, or early nonprimary nonsecondary	42 (36.8)
Unknown duration or late	72 (63.2)
Treatment relative to AKDOC custody	
Completed treatment in AKDOC custody	73 (64.0)
Completed treatment after discharge from AKDOC	15 (13.1)
Began treatment in AKDOC custody but did not complete treatment	13 (11.4)
Untreated or unknown treatment status	13 (11.4)
Completed treatment	
Patients with primary, secondary, or early nonprimary or nonsecondary syphilis (n = 42)	41 (97.6)
Patients with late latent syphilis or of syphilis of unknown duration (n = 72)	47 (65.3)
Newly identified cases in females of reproductive age (15–44 yrs)^§^	37 (100.0)
Pregnant at time of test	1 (2.7)
Not pregnant at time of test	31 (83.8)
Pregnancy status unknown	5 (13.5)

**Abbreviations:** AKDOC = Alaska Department of Corrections; RPR = rapid plasma reagin.

* RPR testing is nontreponemal and detects lipoidal antibodies; when present, although nonspecific, they often indicate a current or past syphilitic infection. If the test is reactive (i.e., detects nonspecific antibodies), the RPR specimen is diluted to obtain a titer, and a treponemal test (i.e., specific to syphilis) is performed on the same sample to confirm syphilitic infection.

^†^ Percentages calculated among those with available data.

^§^ Although CDC defines reproductive age as 15–44 years, all women in this analysis were aged ≥18 years.

^¶^ Although five cases identified outside of AKDOC were not new cases, patients were untreated at the time the cases were identified and had treatment completed while in AKDOC custody.

Among the 126 persons with a reactive RPR who did not have newly identified syphilis, 24 (19.0%) had received a diagnosis of syphilis through routine AKDOC medical services before the opt-out screening program; the remaining 102 (81.0%) had previously identified syphilis outside of AKDOC. All had positive treponemal (confirmatory) testing results.

### Syphilis Staging and Treatment Status

Among the 114 new syphilis cases identified, 42 (36.8%) were staged as primary, secondary, or early nonprimary nonsecondary syphilis; 41 (97.6%) of these patients completed treatment with the indicated single dose of benzathine penicillin or a 14-day course of doxycycline by September 2025 (Figure); the remaining patient left DOC custody before the result was available and could not be located for treatment. The remaining 72 (63.2%) cases were staged as unknown duration or late syphilis, requiring a longer duration of treatment. Among these, 47 (65.3%) patients completed treatment with 3 doses of benzathine penicillin or 28 days of doxycycline, 13 (18.1%) received at least 1 dose of benzathine penicillin or doxycycline but did not complete a full treatment course (partial treatment), and 12 (16.7%) remain untreated or have an unknown treatment status due to inability to be located for treatment completion after AKDOC discharge when test results were returned. Thus, 88 (76.3%) of the 114 patients with newly identified syphilis were known to have completed treatment as of September 2025. Among the 114 newly identified cases, 73 (64.0%) patients completed treatment while in AKDOC custody, 15 (13.1%) completed treatment after discharge from AKDOC, 13 (11.4%) received some treatment in AKDOC custody but are not known to have completed treatment, and 13 (11.4%) remained untreated or had an unknown treatment status as of September 2025. Five cases identified outside of AKDOC were previously untreated and had treatment completed while in AKDOC custody.

## Discussion

Correctional settings in Alaska represent an important opportunity for syphilis detection and treatment. In this analysis, 73 people were successfully treated for syphilis while in AKDOC custody, directly reducing the overall impact of syphilis in adults. The identification and timely treatment of at least one pregnant woman with syphilis might have averted a case of congenital syphilis. Further, additional congenital syphilis cases might have been prevented through this effort in the course of treating syphilis among incarcerated women of reproductive age and their sex partners, although not all patients with new syphilis diagnoses could be located for treatment completion or partner services after AKDOC discharge. Continued efforts to expand screening and treatment in AKDOC so that persons passing through the corrections system too quickly to access regular medical intake or linkage to care at discharge (e.g., treatment completion, partner services, and follow-up care) will be central to optimizing the impact of opt-out screening and treatment.

Because congenital syphilis occurs during pregnancy, reducing the incidence of congenital syphilis might also be addressed by enhanced efforts to meet reproductive health care needs among populations such as incarcerated persons. Increasing the availability of reproductive care in health care settings serving incarcerated persons might assist Alaskans in planning when they want to be pregnant and connect them with effective options for contraception.

### Limitations

The findings in this report are subject to at least four limitations. First, because information on the number or characteristics of persons who opted out of syphilis screening upon entering AKDOC custody was not available, estimation of the proportion of eligible persons who were screened and determination of characteristics associated with opting out was not possible. Second, the population screened might not be representative because it primarily represents persons remaining in AKDOC custody for at least 14 days. Persons briefly in custody, including many of those in pretrial facilities, might not have yet been provided syphilis screening. It is not known whether the incidence of syphilis or the acceptability of syphilis testing differs among persons with shorter stays in custody. Third, the prevalence of syphilis in a screened population is not directly comparable to the annual prevalence of reported cases statewide, as universal opt-out screening does not occur statewide. Finally, pregnancy status was not available for all women of reproductive age with a new syphilis diagnosis; therefore, the true number of congenital syphilis cases averted might be underreported. It is possible that more than one newly identified and treated infection occurred in a woman who was pregnant, but whose pregnancy status was not known.

### Implications for Public Health Practice

CDC recommends conducting opt-out syphilis screening for incarcerated persons based on the local and institutional prevalence of infectious syphilis (*6*). In light of the nationwide increase in syphilis, including congenital syphilis, the lack of timely testing and adequate treatment contributing to nearly 90% of U.S. congenital syphilis cases (*2*), and the relatively high prevalence of syphilis among incarcerated persons, correctional facilities nationwide might benefit from adopting an opt-out screening strategy for syphilis among incarcerated persons.

## Footnotes

* RPR tests have lower sensitivity in the early primary and tertiary stages of syphilis. Because RPR tests do not detect antibodies specific for syphilis, they might yield reactive (i.e., false positive) results for persons with conditions such as autoimmune disease, pregnancy, viral infection, malignancy, or the use of certain drugs or medications, among others.

† During the first 7 months of the analysis period in this report (October 2023–May 2024), 2,330 persons were tested for syphilis in AKDOC, or 85.5% of the 2,633 persons who were in AKDOC custody for ≥14 consecutive days during that period. Testing rates for the complete period were not available at the time of this report but are estimated to be similar.
